# Multi-Modal Fusion Emotion Recognition Method of Speech Expression Based on Deep Learning

**DOI:** 10.3389/fnbot.2021.697634

**Published:** 2021-07-09

**Authors:** Dong Liu, Zhiyong Wang, Lifeng Wang, Longxi Chen

**Affiliations:** School of Information Engineering, Shandong Youth University of Political Science, Jinan, China

**Keywords:** deep learning, multimodal fusion, voice, expression, emotion recognition, long short-term memory, LibSVM classifier

## Abstract

The redundant information, noise data generated in the process of single-modal feature extraction, and traditional learning algorithms are difficult to obtain ideal recognition performance. A multi-modal fusion emotion recognition method for speech expressions based on deep learning is proposed. Firstly, the corresponding feature extraction methods are set up for different single modalities. Among them, the voice uses the convolutional neural network-long and short term memory (CNN-LSTM) network, and the facial expression in the video uses the Inception-Res Net-v2 network to extract the feature data. Then, long and short term memory (LSTM) is used to capture the correlation between different modalities and within the modalities. After the feature selection process of the chi-square test, the single modalities are spliced to obtain a unified fusion feature. Finally, the fusion data features output by LSTM are used as the input of the classifier LIBSVM to realize the final emotion recognition. The experimental results show that the recognition accuracy of the proposed method on the MOSI and MELD datasets are 87.56 and 90.06%, respectively, which are better than other comparison methods. It has laid a certain theoretical foundation for the application of multimodal fusion in emotion recognition.

## Introduction

Under the background of the era of big data, with the continuous development of artificial intelligence, the fields of human–computer emotional interaction, emotion recognition, and human–computer interaction have gradually become research hotspots (Kim and Lee, 2021). As the foundation of human–computer emotional interaction research, emotion recognition affects the development of artificial intelligence technology. At the same time, due to the integration of knowledge of multiple disciplines, the development of emotion recognition research has also led to the rapid development of other disciplines. At present, the research of emotion recognition is mainly concentrated in the field of monomodal emotion recognition such as text, speech, and image (Rao et al., 2019). Although unimodal emotion recognition has made many breakthrough achievements, with the passage of time, unimodal emotion recognition has also exposed some problems. It cannot fully describe a certain emotion of the user at the moment, and using multiple modal features to describe a certain emotion together will be more comprehensive and detailed (Wei et al., 2020; Zhang J. H. et al., 2020). In addition, there are certain information associations within and between different single modalities. When a certain modality contains less emotional information, the rest of the modality information can provide a supplement for the emotion classification task (Mou et al., 2019; Zhao et al., 2019). Therefore, multi-modal fusion emotion recognition is the key research direction of human–computer interaction.

Generally speaking, emotion recognition includes the steps of data information acquisition, preprocessing, emotion feature extraction, and classification (Ma et al., 2019). In order to make full use of the existing modal data, while capturing the relevant information within and between the modalities, multi-modal fusion is gradually developed and applied in emotion recognition. The traditional multi-modal emotion recognition method will increase the calculation time and space cost, and the recognition effect will be disturbed. And traditional machine learning cannot directly use the high-dimensional data of each modal itself (Rossi and Ruocco, 2019). At the same time, how to evaluate the internal characteristics of each single modality, remove redundant information and noise data, and reduce the time and space consumption of emotion recognition calculation are all problems to be solved urgently (Du et al., 2019; Barabanschikov and Suvorova, 2020).

In order to make better use of the single-modal emotion characteristics and achieve high-accuracy emotion recognition, a deep learning-based voice expression multi-modal fusion emotion recognition method is proposed.

The rest of this paper is organized as follows. The third section introduces the related research in this field. The third section introduces the speech expression multimodal emotion recognition model. In the fourth section, in order to demonstrate the performance of the proposed method, a series of experiments are carried out using the multimodal opinion level sentient intensity corpus (MOSI) data set and Multimodal EmotionLines Dataset (MELD) data set to verify the performance of the proposed method. The sixth part summarizes the whole paper.

## Related Research

In human–computer interaction, the accurate expression and recognition of emotions require multiple media. The amount of information covered by multiple modalities is greater than the amount of information covered by single modalities, which can improve the robustness of emotion recognition (Elleuch and Wali, 2019). Multi-modal emotion recognition technology fully considers the interaction between text, speech, image, and other modalities. Comprehensive use of information from multiple information sources to perform emotion recognition on target tasks (Huddar et al., 2020). At present, the modalities used in multi-modal emotion recognition mainly include text, voice, expression, video, and biological signals. Most of them are free combinations of different modalities to complete the corresponding emotion recognition task (Lovejit et al., 2019).

At present, many domestic and foreign researchers are devoted to the research of multi-modal fusion emotion recognition. But most of them use traditional feature extraction methods, such as Hidden Markov Model (HMM) and Gaussian mixed Model (GMM) models (Andy and Kumar, 2020). Zhang Z. et al. (2020) aimed at the detection and classification of microcalcification clusters by computer-aided diagnosis systems, using an adaptive GMM model to extract spatial distribution statistical features, which has high accuracy. However, the feature extraction process is relatively simple, and the feature extraction for complex scenarios has not been verified. Engin and Cavusoglu (2019) proposed a multi-resolution curvelet transform-based symbiosis and Gaussian mixture feature rotation invariant texture representation model for image retrieval and classification. However, the classification process is more complicated, which affects the recognition efficiency to a certain extent. Vivekanandam and Babu (2019) proposed an HMM model for facial expression classification and recognition in videos, and improved random features by Gaussian kernel method. But the emotion recognition process is mostly based on the ideal environment, and less consideration is given to influencing factors. Choras and Zhou (2019) proposed an emotion recognition method based on auditory characteristics. Gammatone frequency cepstrum coefficient and GammChirp frequency cepstrum coefficient are used as characteristic methods. And use the HMM model as a classifier to achieve more accurate emotion recognition. But these models can only model limited contextual information. The characteristics of slow changes in human emotions and strong dependence on contextual information cannot be fully utilized (Srikanth and Mohan, 2020). Compared with traditional feature extraction methods, deep neural networks can effectively reveal the internal structure hidden in the data, and efficiently extract abstract features useful for emotion recognition (Lovejit et al., 2019).

Taking into account the inherent relevance between different modal information, the emotional characteristics of different modalities, such as text and sound, but with strong relevance can be merged accordingly. Get a more effective feature representation that can be used for emotion recognition (Bc and Prakash, 2020). The main purpose of deep learning is to automatically obtain effective feature representations from data. Through multiple feature transformations, the original data features can be transformed into effective feature representations. Finally input into the classification model to get the recognition result (Li et al., 2021). As the basic model of deep learning, Convolutional Neural Networks (CNN), combined with network models such as Recurrent Neural Network (RNN) and Long Short Term Memory Network (LSTM), have been widely used in emotion recognition. And produced many corresponding optimization models, all of which have achieved good research results (Liu and Zhou, 2020). Hossain et al. (2020) focuses on feature extraction with high discrimination in the process of action recognition, and proposes a feature coordination recognition strategy that enhances ResNet101 and successive entropy supervision training. Realize high-accuracy video action recognition. In order to better obtain the emotional features in the speech signal, Jiang et al. (2019) proposed a parallel convolutional RNN with spectral features for speech emotion recognition. Among them, multiple high-level features are fused and then batch normalized, and the SoftMax classifier is used to classify emotions, which achieves better emotion recognition. However, the effect of single mode on emotion recognition is still weak. Rajesh and Nalini (2020) proposed a RNN-based emotion recognition method for musical instruments, which has better performance than the benchmark machine learning classification algorithm. However, the application environment of this method is relatively special and lacks certain universality. Li et al. (2020) proposed an emotion recognition framework based on RNN with parameter deviation. By fusing the coordinates, angles and angular velocity of human joints, expressions are captured for emotion recognition. The combined structure of various modes in subconscious behavior is proposed to improve the classification performance of the proposed method. In multi-modal emotion recognition tasks, deep neural network models can also be used for feature learning of fused multi-modal data (Pan et al., 2020). The deep neural network imitates the process of human neural tissue processing information, so that the original data is in the deep network structure, and then used in the feature engineering of multi-modal sentiment analysis (Luo et al., 2020). Therefore, a deep learning-based voice expression multimodal fusion emotion recognition method is proposed to achieve high-efficiency and high-accuracy emotion recognition.

## Proposed Multi-Modal Emotion Recognition Method for Speech Expression

### Overall Model Architecture

**Figure 1:**
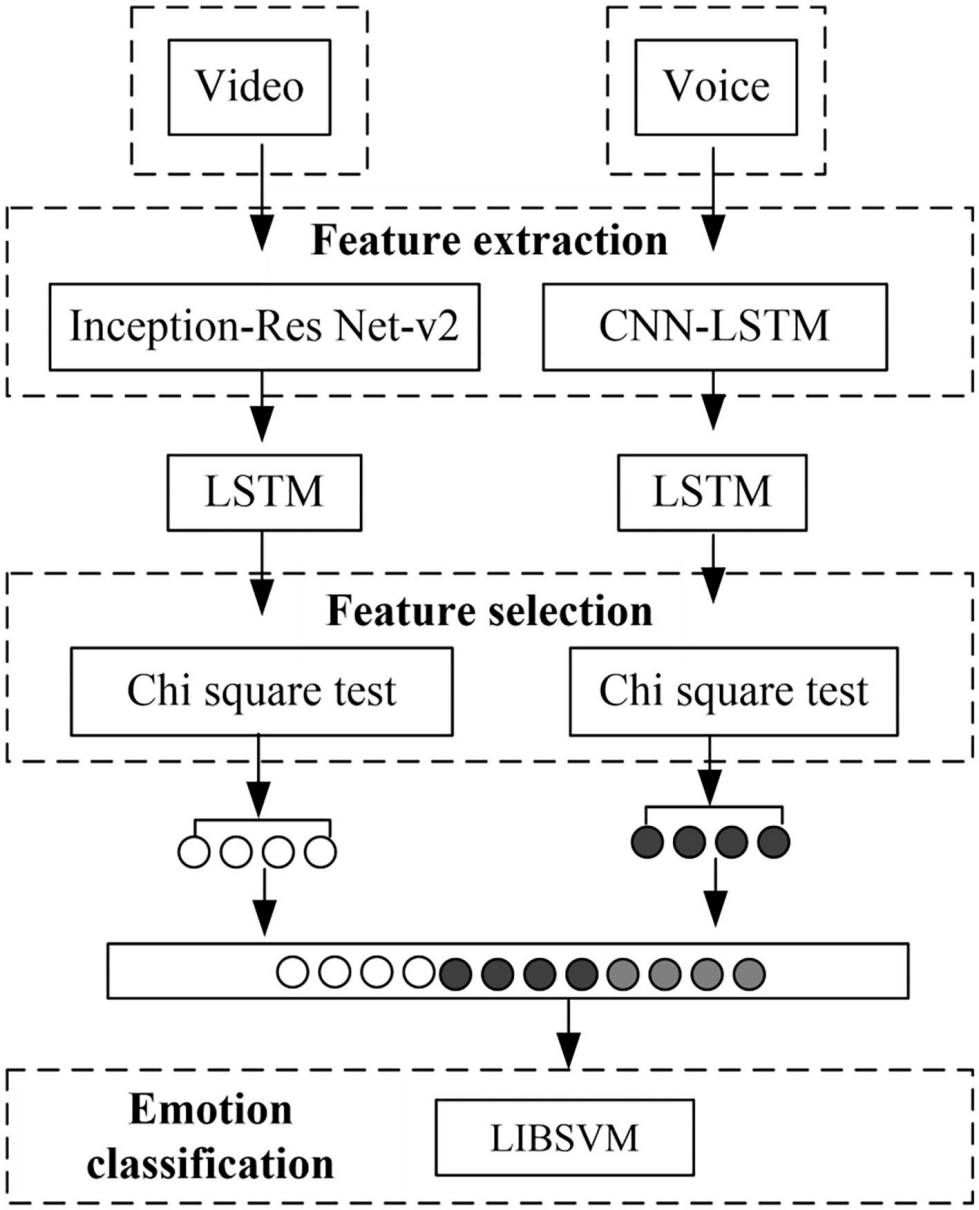
Multimodal emotion recognition model based on deep learning.

The overall architecture of the multi-modal fusion emotion recognition model for speech expressions based on deep learning is shown in Figure 1. It is mainly divided into three parts: feature extraction module, feature selection module, and emotion classification module.

The original data on social platforms cannot be directly used for emotion classification tasks, so the original modal needs to be transformed. The feature extraction module is the basis of the entire multi-modal emotion recognition model. It is mainly used to process the input modal information and turn it into features that can be used by the model (Choi and Song, 2020). In the feature extraction module, different feature extraction schemes are set for different single modes. The voice uses the convolutional neural network-long and short term memory (CNN-LSTM) network, and the video uses the Inception-Res Net-v2 network to extract the corresponding feature data.

Multi-modal emotion data tries to portray the current emotion of a user from different angles. On the one hand, at a certain moment, different modalities occupy different weights in the description of emotions, which will cause data redundancy and data noise. On the other hand, single-modal features may also cause data redundancy and noise information in the feature extraction process. If data redundancy and data noise are not processed, it will not only increase the difficulty of model training, but also cause waste of resources. When the scale of the data set is relatively large, there are more corresponding monomodal features extracted. When multiple modalities are merged, it can even lead to a “dimension disaster.” To this end, a feature selection module is added to denoise the features of each mode.

Within the single mode, there are correlations between features, and the LSTM structure can be used to capture the time dependence on the time series. At the same time, there are also information associations between the various modalities. These associations can be used as hidden emotional clues to help the final emotional decision-making. When one modal information is sparse, or when two or more emotions are inconsistent, LSTM can capture the relationship between different modalities and make emotion recognition more accurate. The extracted single-modal features are sent to the LSTM unit to capture the interdependence within the modal. After the feature selection process, the monomodal features are spliced to obtain a unified fusion feature. Then the LSTM structure is added to capture the interdependence between modalities. Finally, the output data of LSTM is used as the input of the classifier LIBSVM to make the final emotional decision.

### Speech Signal Feature Extraction

**Figure 2:**
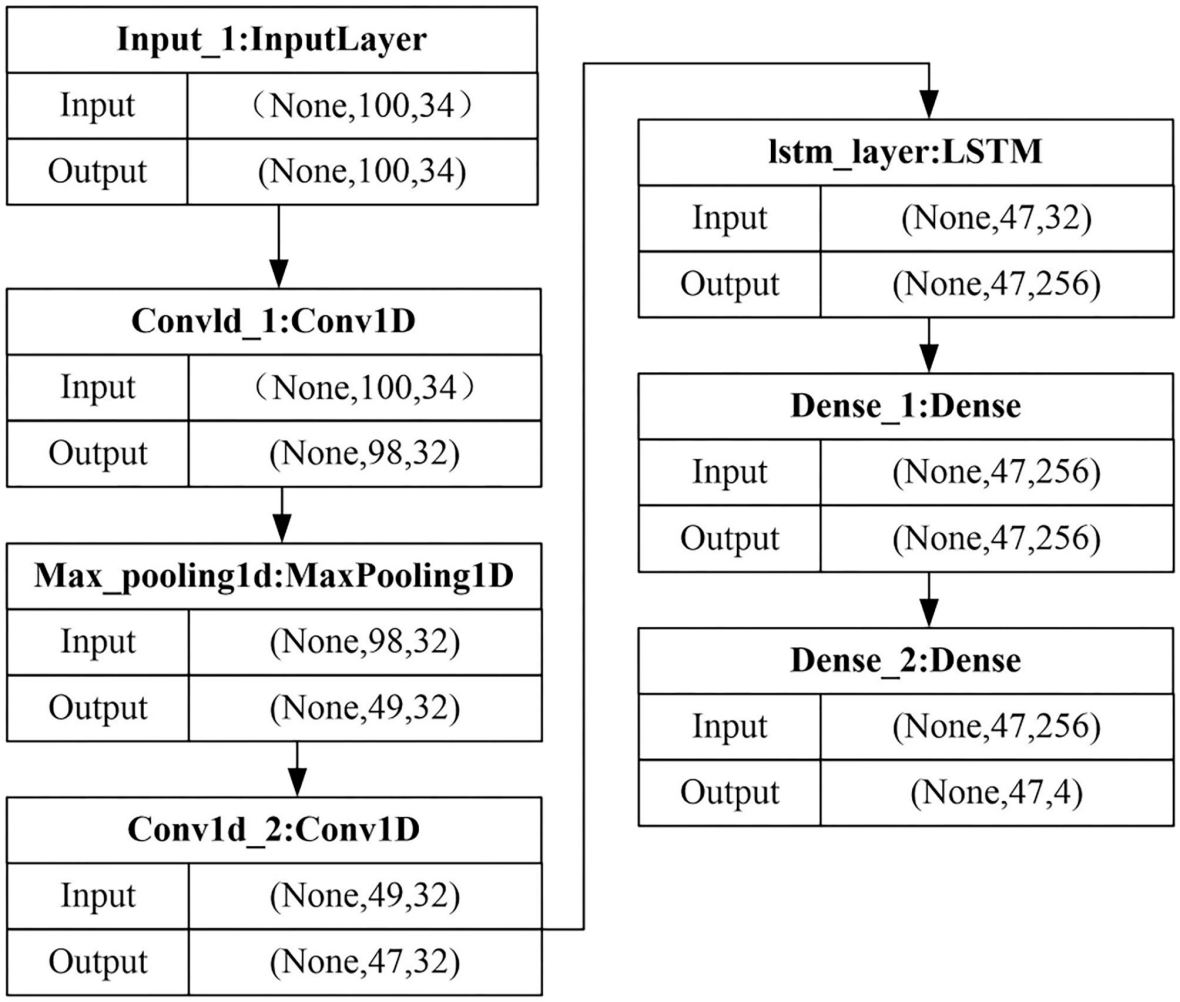
Model flow graph of CNN-LSTM speech feature extraction.

Speech is a non-linear time sequence signal, which is closely related to time (Asada et al., 2020). At the same time, the LSTM network is suitable for the extraction and learning of speech features. In context-sensitive information modeling, it is helpful to learn the temporal information of speech emotion features. However, there is no intermediate non-linear hidden layer in the LSTM network, which leads to an increase in the hidden state factor. For this reason, the proposed method uses the fusion network of CNN and LSTM to learn the deep abstract acoustic emotional features of speech. The speech feature extraction process based on CNN-LSTM is shown in Figure 2.

In the CNN-LSTM network, the structural characteristics of the CNN network model are combined, and an LSTM layer is added on the basis of it. Use the convolutional layer to transform the input data, and then input it into the LSTM layer for re-learning. Among them, the convolution part is composed of two convolution layers and a maximum pooling layer. The parameters of the convolutional layer are the same as those in the CNN network, which is 32 × 3, and the activation function is a Rectified Linear Unit (ReLU). The output dimension of the LSTM layer is set to 256, and the Recurrent Dropout parameter is set to 0.2. This parameter represents the ratio of discarded linear transformations in the recursive state. The output layer of all models is the Dense layer, and the output dimension is the number of types of sentiment classification.

### Facial Expression Feature Extraction

Usually the amount of data in the video is very large. Find the face contour of each frame of the video, and reduce the size of the frame image by cropping the face contour picture, which is conducive to subsequent data processing and feature learning (Brito et al., 2020; Seo and Hong, 2020). The proposed method expresses the emotional information in the video by extracting short time Fourier transform (STFT) features and using the Inception-Res Net-v2 network to extract deep features. Short time Fourier transform is a general tool for speech signal processing. It defines a very useful class of time and frequency distribution, which specifies the complex amplitude of any signal varying with time and frequency. In fact, the process of calculating short-time Fourier transform is to divide a longer time signal into shorter segments of the same length, and calculate Fourier transform (Fourier spectrum) on each shorter segment.

**Figure 3:**
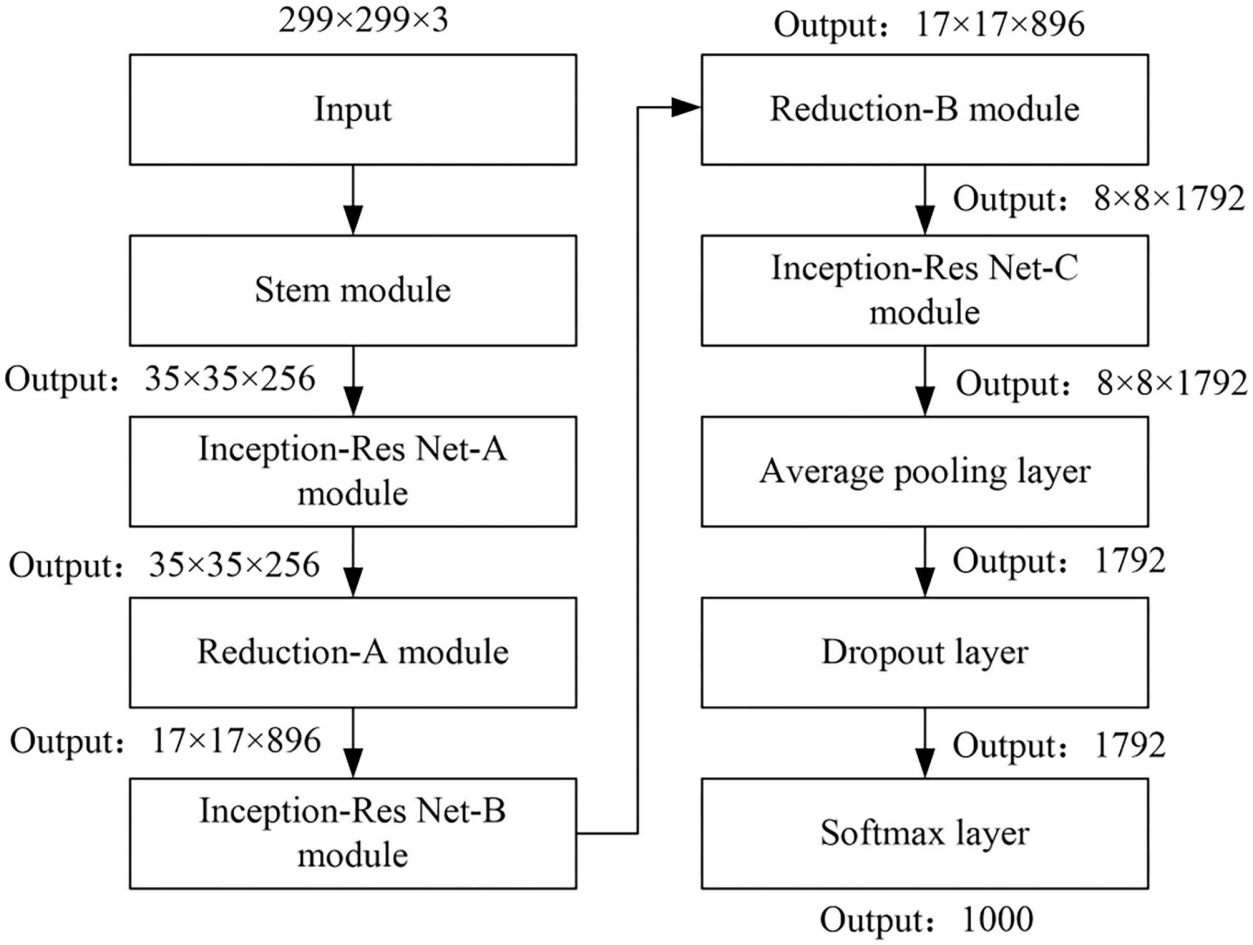
Network mode flow diagram of Inception-Res Net-v2.

Inception-Res Net-v2 is a CNN, which was constructed by Google in 2016 by introducing a residual network (ResNet) model on the basis of the Inception model. It has an accurate recognition effect on very similar objects. This network is a variant of the early Inception V3 model. It draws on the residual connection idea in the Microsoft ResNet model, so that the neural network can be trained deeper. By using multiple 3 × 3 convolutions instead of 5 × 5 and 7 × 7 convolutions, the Inception module can be extremely simplified. To a large extent, the computational complexity and parameter dimensions are reduced, and the training speed of the network is accelerated. But compared with other networks, the space complexity of this algorithm is higher. The flow diagram of the Inception-Res Net-v2 network mode is shown in Figure 3.

The Inception-Res Net-v2 network is mainly composed of six modules including stem, Inception-Res Net-A, Reduction-A, Inception-Res Net-B, Reduction-B, and Inception-Res Net-C. The output vectors of the Convolution layer, Avg Pool layer, Dropout layer, and Fully Connected layer that are finally connected by the network can be regarded as the depth features of the samples obtained after each layer of learning. By classifying and comparing the features output by Inception-ResNet-v2 in the Convolution layer, Avg Pool layer, Dropout layer, and Fully Connected layer, the fully connected layer features with better recognition effect are selected as the extracted deep video features.

### Feature Selection

**Figure 4:**
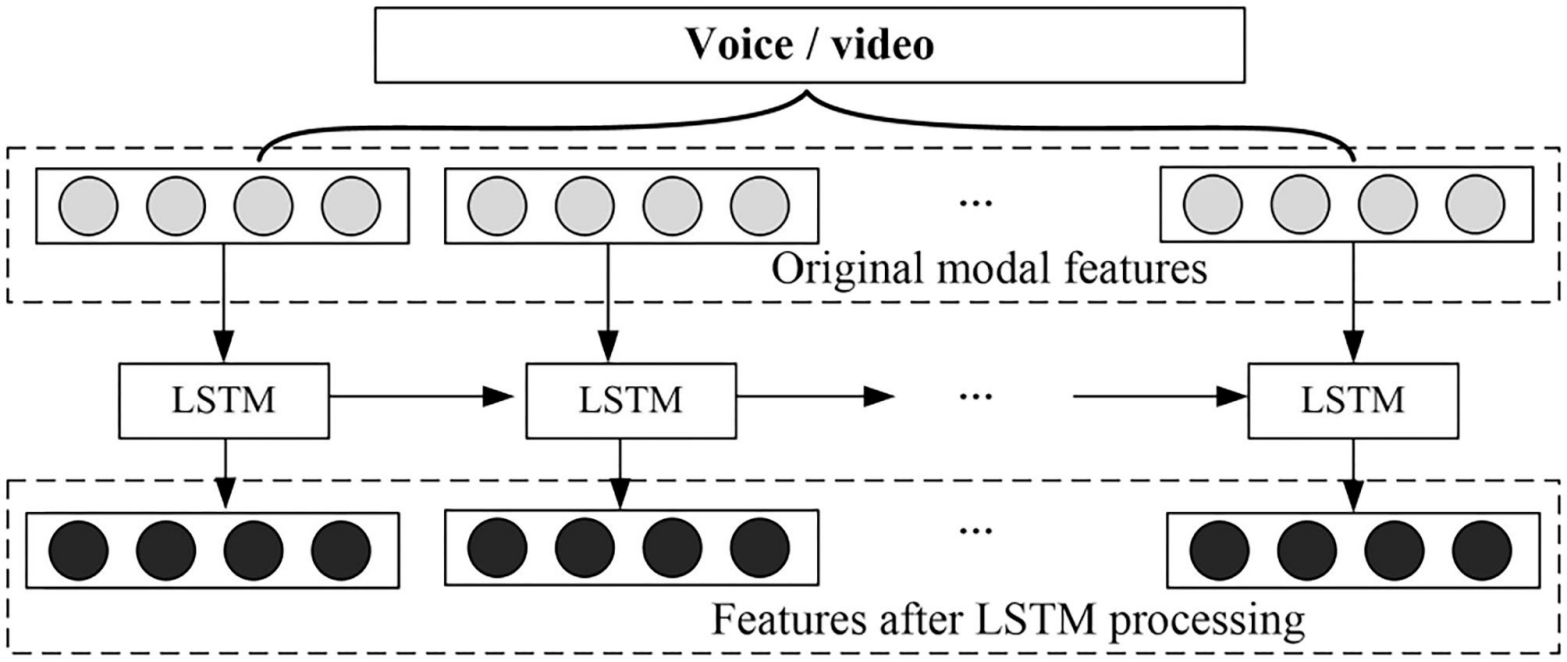
RNN is used to capture modal internal dependencies.

In order to capture the dependence between the internal features of each mode, the LSTM structure of the RNN is used (Eromski et al., 2020). Similarly, there is not only a correlation between the internal features of a single mode, but also interdependence between the features of different modes. When the features of one mode are sparse, the information contained in the other mode can help emotional decision-making (Du et al., 2020). In this regard, the proposed method also uses the LSTM structure to obtain the dependence between the various modes, and the specific structure is shown in Figure 4.

Assuming that the monomodal is *h*-dimensional, the features in the monomodal can be represented by the feature vector $x_{i,t}\in R^{h}$, and *t* represents the *t*-th sentence in the video *i*. For a video, you can collect all the vectors it contains to obtain the vector matrix $X_{i}=\left[x_{i,1},x_{i,2},\cdots ,x_{i,L}\right]\in R^{L_{i}\times h}$. *L*_*i*_ represents the total number of sentences in the video, and the matrix *X*_*i*_ can be used as the input of LSTM.

In addition, in order to improve the generalization ability of the emotion recognition method and reduce the amount of calculation, the proposed method uses a filtering method for feature selection. Select feature subsets based on the general features of the data, and use statistical methods to assign feature scores to features. Then these features are sorted in descending order according to the scores, and the features with higher rankings are kept in the feature subset. And filter out irrelevant feature groups based on the feature subset.

At present, the more widely used methods include chi-square test and information gain. The proposed method uses chi-square test for feature selection, which is used to indicate the correlation between the feature and the corresponding class. Chi-square test is used to measure the dependency between features and classes. A higher score means that the relevant class is more dependent on a given feature. Therefore, features with lower scores have less information and should be deleted. Assuming that the feature is independent of the final classification value, the chi-square test can be defined as:

(1) $$\begin{array}{r} Chi\left(f,c_{i}\right)\;=\;\frac{N\times {\left(AD-BC\right)}^{2}}{\left(A+C\right)\times \left(B+D\right)\times \left(A+B\right)\times \left(C+D\right)}\\ \;Chi_{\max}\left(f\right)=max\left(Chi\left(f,c_{i}\right)\right) \end{array}$$

Where, *A* is the number of documents that have feature *f* and belong to category *c*_*i*_; *B* is the number of documents that have feature *f* but do not belong to category *c*_*i*_; *C* is the number of occurrences of *c*_*i*_ without *f*; *D* is the frequency when neither *c*_*i*_ nor *f* appears; *N* is the total number of instances in the document set, *N* = *A*+*B*+*C*+*D*. If *f* and *c*_*i*_ are independent, the *Chi* statistic will be zero.

Redundant information and noise information are filtered out within the single mode. The chi-square test directly selects a subset of features from the original features, uses the chi-square value to sort the available features, and automatically filters out those features whose scores are lower than a predetermined threshold.

### Feature Classification

**Figure 5:**
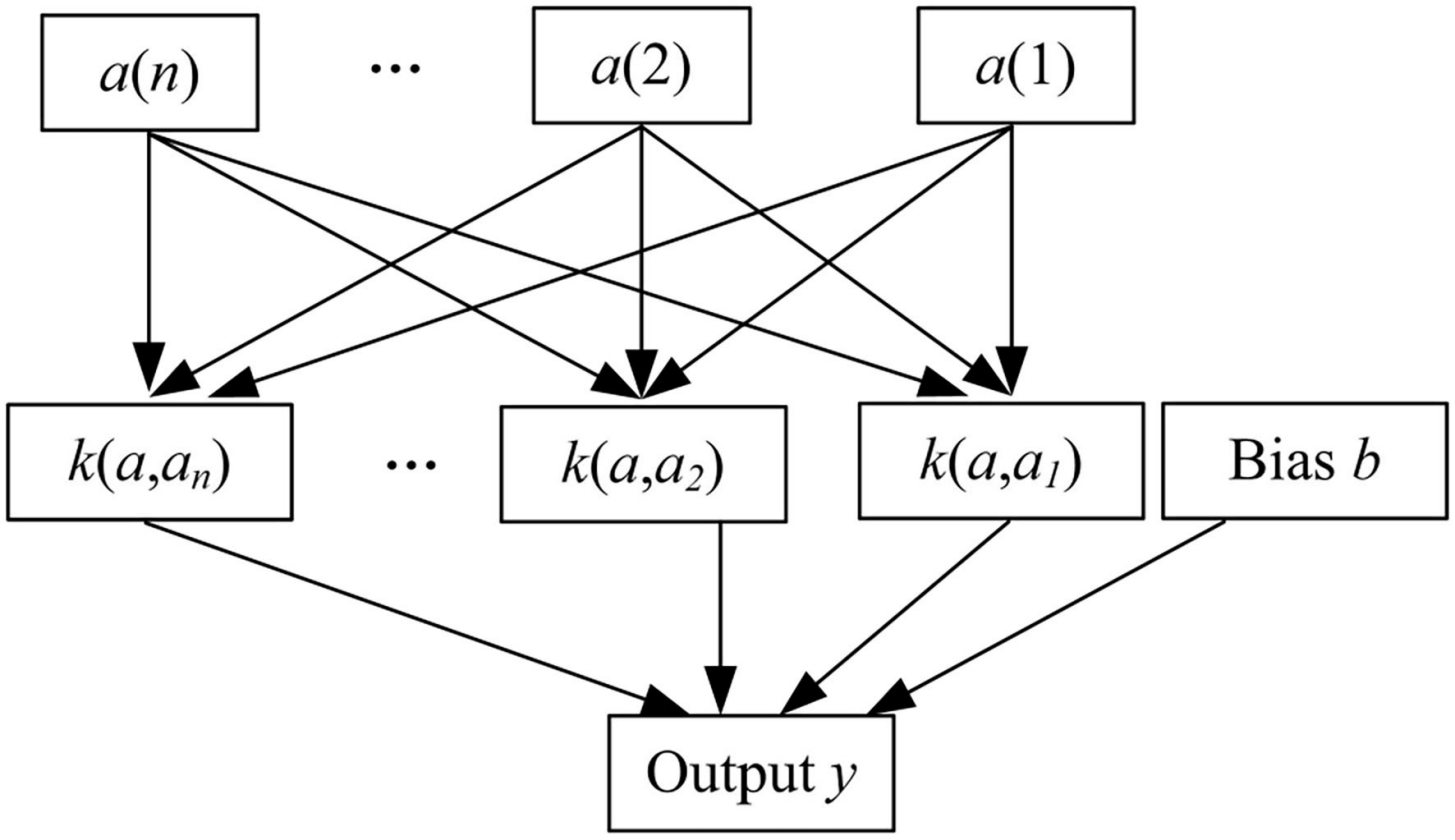
LIBSVM architecture.

The two feature values of speech and facial expressions are fused together as the final feature value of the multi-modal signal (Kuznetsov and Oseledets, 2019). The proposed method uses LIBSVM as the classifier to perform sentiment classification. It is a further improvement on the SVM and a supplement to some parameters of the original SVM. LIBSVM is usually used to solve two classification problems, by establishing a hyperplane, as far as possible to distinguish between positive examples and negative examples. Its system structure is shown as in Figure 5.

Among them, *k*(*a, a*_*i*_) represents the kernel function, and its role is to map data from low-dimensional space to high-dimensional space, so as to solve the problem of inseparable data in low-dimensional space. The offset *b* represents the offset of the hyperplane relative to the origin. Under LIBSVM, in addition to the kernel function, there is also an important parameter penalty factor ϑ, which represents the degree of emphasis on outliers. When ϑ is larger, the emphasis on outliers is higher, and the existence of errors is not allowed. When ϑ is close to 0, the existence of error is basically not considered, that is, the correctness of the classification result is not considered. Construct the decision function for the test sample through the training sample and the corresponding label, kernel function and penalty factor ϑ.

When performing emotion classification, an emotion recognition result will eventually be obtained for each channel participating in emotion recognition. At this time, each channel can be regarded as a set of separate multi-modal signals, that is, a separate LIBSVM classifier is formed. For the classification results generated by each LIBSVM classifier, the decision-level fusion is performed. The proposed method uses the fusion of multiple classifiers based on fuzzy integral. Not only can the results obtained by each LIBSVM classifier be fused during classification, but also the importance of each LIBSVM classifier in the fusion process can be reflected. Fuzzy integral uses fuzzy measure as the weight of each LIBSVM classifier, and considers the relationship between each LIBSVM.

## Experiment and Analysis

In order to demonstrate the performance of the proposed method, a series of experiments were performed using the MOSI dataset and the MELD dataset. At the same time, the classification cross entropy is used to train the LSTM structure, and the loss function is defined as:

(2) $$\begin{array}{r} loss\;=\;-\frac{1}{\sum_{i=1}^{M}L_{i}}\overset{M}{\underset{i=1}{\sum }}\overset{L_{i}}{\underset{j=1}{\sum }}\overset{C}{\underset{c=1}{\sum }}y_{i,c}^{j}\log_{2}{ŷ}_{i,c}^{j} \end{array}$$

Where, *M* is the total number of videos. *L*_*i*_ is the number of sentences in the *i*-th video. $y_{i,c}^{j}$ is the original output category of the *j*-th sentence of the *i*-th video. ${ŷ}_{i,c}^{j}$ is the recognition output of the *j*-th sentence of the *i*-th video.

When the video length is different, the “zero padding” method is adopted to avoid the increase of noise in the network. At the same time, the training set is divided into training set and verification (70/30%) for parameter adjustment. During the training of the LSTM network structure, set the dropout to 0.99, the fully connected layer to 300, the number of iterations to 500, and the mini-batch size to 50 to avoid over-fitting.

### Dataset

#### MOSI Dataset

The MOSI data set randomly collects 93 popular update videos from You Tube. Mainly the speaker expresses his or her views on a certain movie. The length of the video is usually kept at 2–5 min. Most speakers in the MOSI data set are between 20 and 30 years old. Although the speakers come from different ethnic backgrounds (for example, Caucasian, African American, Hispanic, Asian), all speakers are expressed in English. Each video is divided into multiple clips, and each clip contains one or more sentences expressing opinions. Five emotional annotators annotated these clips, resulting in 2,199 subjective fragments and 1,503 objective fragments. The range of emotional polarity is [−3, +3], corresponding to strong positive (+3), positive (+2), weak positive (+1), neutral (0), weak negative (−1), negative (−2), strong negative (−3). Some image examples of the MOSI dataset can be found here: https://github.com/A2Zadeh/CMU-MultimodalSDK.

At the same time, some statistical data parameters of the MOSI dataset are shown in Table 1.

**Table 1:** MOSI statistics parameters.

**Data item**	**Parameters**
Number of video segments	3,702
Number of emotion segments	2,199
Total number of videos	93
Average length of emotion segment	4.2 s
Average number of words per emotion segment	12
Average number of emotion segments in video	23.2
Total number of different speakers	89

In the experiment, the average value of these annotations is regarded as the emotional polarity, and only two categories (positive and negative) are considered. The training set and the validation set are composed of the first 61 individuals and 1,435 sentences in the data set. The test set consists of the remaining 32 speakers and 764 sentences.

#### MELD Dataset

The MELD data set is a multi-modal data set, including face pictures and corresponding character voices. The face picture comes from the video frame, and the voice corresponds to the video frame. All face pictures and voice data are extracted from five seasons of TV series. The MELD data set includes five seasons S01E03 (season 3), S04E04, S05E05, S07E07, and S10E15. In general, MELD contains about 120,000 face images, which correspond to six characters: Rachel, Monica, Phoebe, Joey, Chandler, Ross. For each character, there are approximately 27,000 face pictures. Similarly, the speech fragment corresponds to a sequence of face pictures. The situation of the face picture can be found here: https://affectivemeld.github.io/.

There are many influencing factors such as angle rotation, occlusion, blur, and poor light in face pictures. As shown in the first row, these influencing factors make face recognition difficult. And the long-lasting poor light makes it impossible to see the human face clearly. For recognizing people, the inability to extract useful facial features makes recognition difficult. Similarly, facial expression changes have always been one of the most influential factors in face recognition tasks. At the same time, MELD has 10 seasons, that is, each actor in the play has gone through 10 years. During the 10 years, as the age changes, the recognition rate is likely to decrease. Chandler's face changed from a young and young boy to an uncle, looking more calm. If you don't observe these pictures carefully, you won't find that these faces belong to the same person. These are the key factors that affect face recognition in the testing phase. Therefore, for each character, the least face picture is Rachel, with 2,600 face pictures. Similarly, Ross has the most face pictures at 6,800. Other roles are between 2,600 and 6,800, and the distribution has a certain imbalance.

### Parameter Discussion

**Figure 6:**
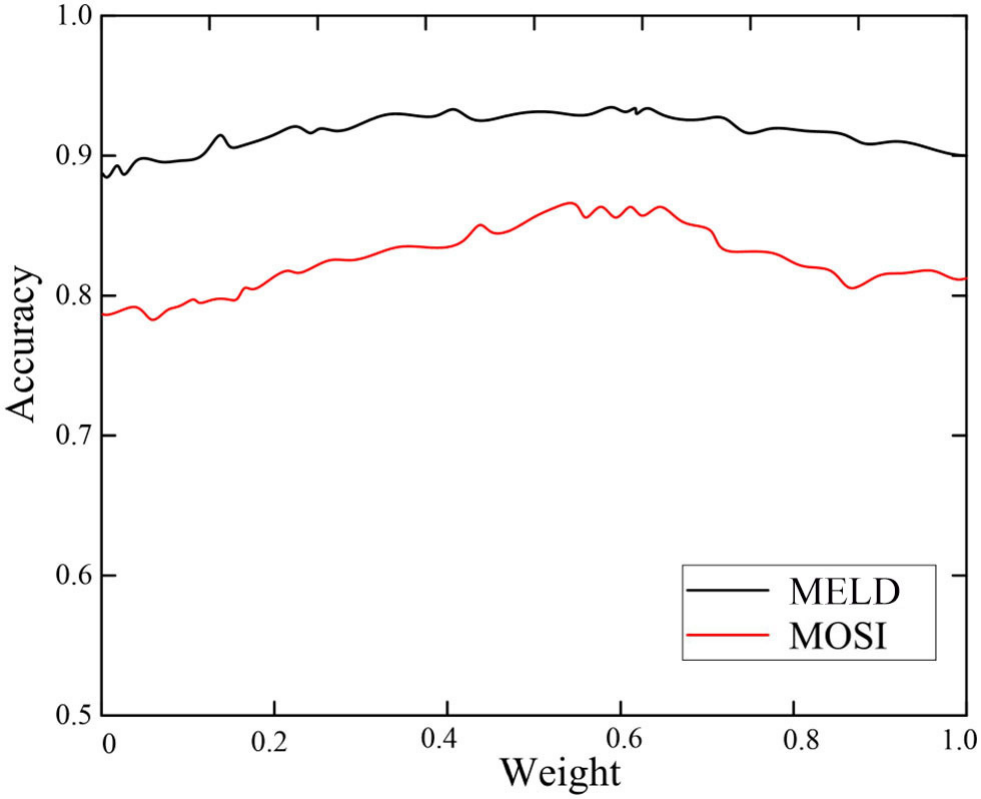
Selection of network weight.

In order to explore the influence of network weight selection in the LSTM network on the recognition results, the relationship between the weight and the emotion recognition accuracy on the MOSI and MELD data sets is shown in Figure 6.

It can be seen from Figure 6 that the emotion recognition accuracy rate increases first and then decreases as the network weight increases. Therefore, whether it is the MOSI dataset or the MELD dataset, the recognition accuracy is the highest when the weight is about 0.57, which is 88 and 92% on the two datasets, respectively.

### Recognition Performance of Each Layer of Inception-Res Net-V2 Network

In the deep feature extraction part of the single-mode video signal, in order to be able to select better output features in the Inception-Res Net-v2 network, the output features of each layer are classified. Compare the recognition performance of each layer, as shown in Table 2.

**Table 2:** Recognition rate of output features in each layer of Inception-Res Net-v2 network.

**Output features in each layer**	**Recognition rate (%)**
Convolution layer features	66.09
AvgPool layer features	63.84
Dropout layer features	79.18
Fully connected layer features	85.75

It can be seen from Table 2 that the output feature recognition accuracy of the fully connected layer is the highest, which is 85.75%. It proves that compared with other layer features, the fully connected layer has the strongest ability to express emotions.

### Network Performance

**Figure 7:**
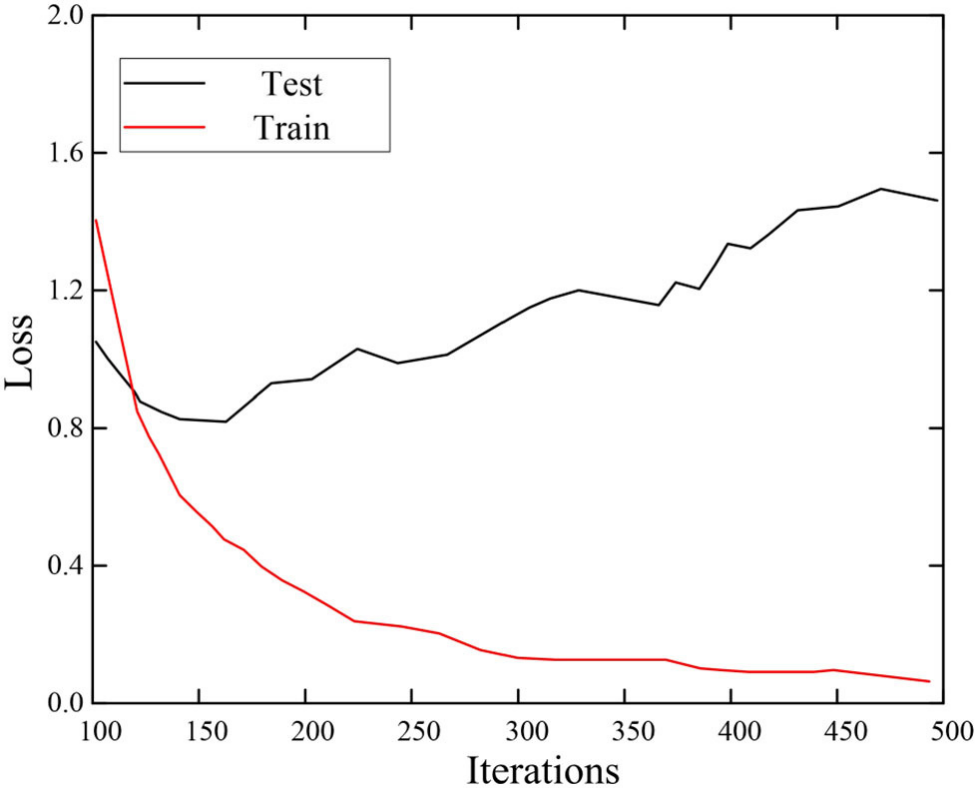
Identify the change of model loss function.

The configuration of the loss function is one of the important steps to ensure that the model runs in the expected way. The two important parameters are the recognition output and the true output of the model. If the identification value is far from the true value, the loss value will also be large. During model training, this loss value will be propagated through the network, and the weight of the network will change accordingly. On the contrary, when the recognized situation is very close to the real situation, the weight of the network will not change much. It shows that the network performance has reached a relatively good condition at this time, and the value of the loss function at this time will be relatively small. Therefore, the loss function is also one of the model evaluation indicators. On the training set and test set, the loss function changes are shown in Figure 7.

It can be seen from Figure 7 that as the number of iterations increases, the loss function value of the training set continues to decrease, and finally stabilizes, with a value close to 0.22. However, due to the influence of various factors on the test set, the value of the loss function fluctuates greatly, but its value is still lower than 1.4. It can be proved that the proposed model has better performance in emotion recognition.

### Chi-Square Test Model Performance

**Figure 8:**
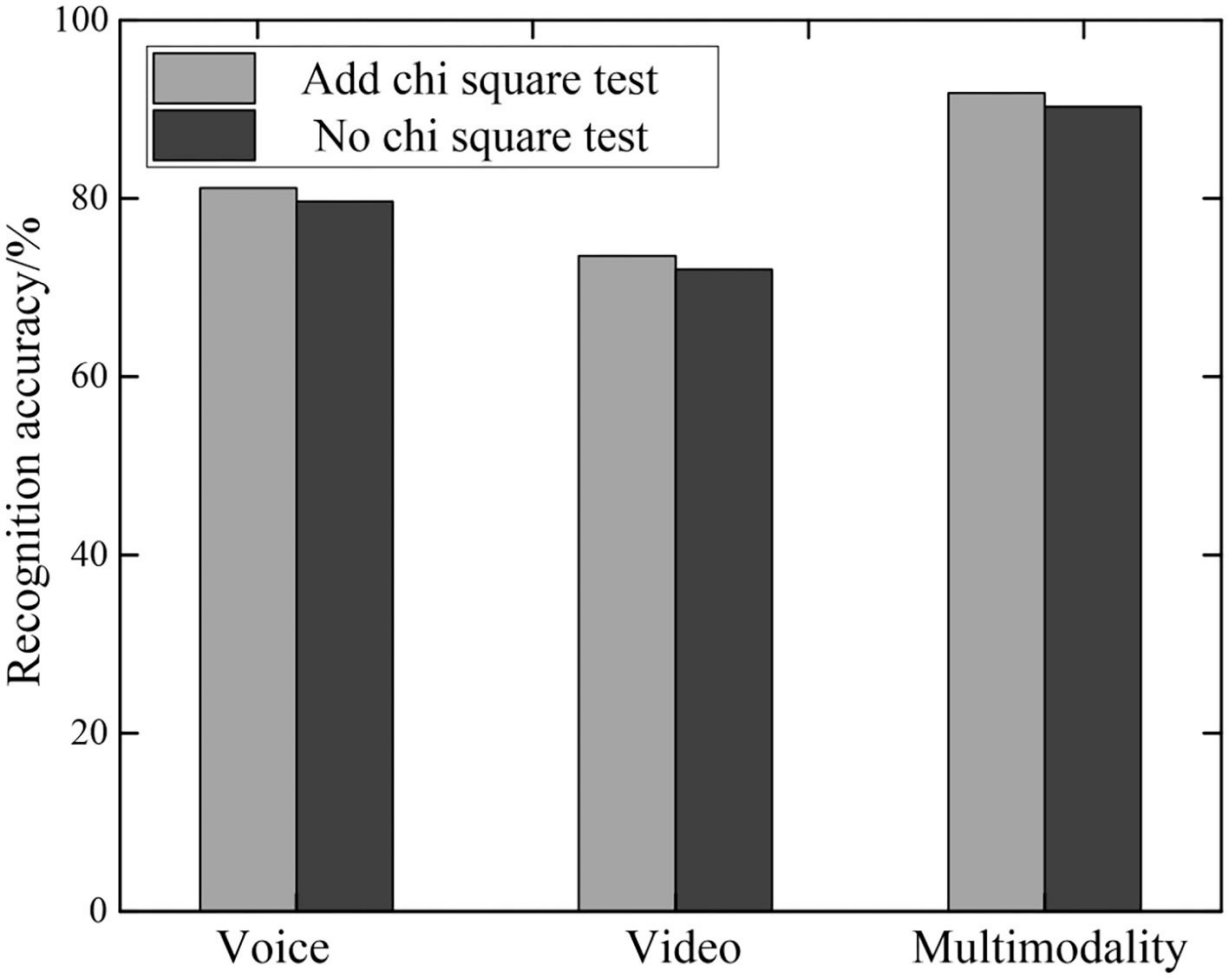
Multimodal emotion recognition with and without chi-square test.

The proposed method first uses the chi-square test method to remove redundant information and noise information. Then perform emotion recognition on the monomodal features after feature selection processing. Finally, the two modalities are combined for multi-modal emotion recognition. In order to test the necessity of using chi-square test to eliminate redundant information and noise information in the multi-modal emotion recognition method, a set of experiments was set up to compare the performance of the model with and without chi-square test feature selection. The result is shown in Figure 8. Among them, through parameter tuning, it can be found that the recognition effect is best when the top 50% of the chi-square value is retained. In order to remove the redundant information and noise data generated in the process of monomodal feature extraction, the proposed method uses the LSTM structure to obtain the internal information dependence of each monomodal. In addition, the chi-square test feature selection method is added to remove redundant features and noise.

It can be seen from Figure 8 that the accuracy of emotion recognition of multi-modal fusion is significantly higher than that of single-modal emotion recognition. Among them, the recognition accuracy of multi-modality is about 90%, while that of single-modality is <80%. At the same time, the recognition accuracy of the model with the chi-square test is significantly higher than that of the model without the chi-square test. Taking multi-modality as an example, with the addition of chi-square test, the accuracy of emotion recognition has increased by 3%. This shows that it is meaningful to use the chi-square test to remove redundancy and noise from multi-feature information.

### Recognition Accuracy of Different Emotions

There are certain shortcomings in the single use of voice signals or video signals for emotion recognition. Therefore, it is necessary to perform multi-modal signal fusion. For example, the low recognition rate of disgusting emotions in video signals needs to be compensated by using voice information. Table 3 shows the recognition accuracy rates of happy, anger, disgust, sad, fear and other emotions in different modalities.

**Table 3:** Accuracy of emotion recognition based on different signal features.

	**Recognition accuracy (%)**		
	**Voice**	**Video**	**Multimodality**
Happy	81.25	83.17	90.89
Angry	87.83	80.55	91.26
Disgust	58.36	62.49	76.72
Sad	84.29	82.45	87.13
Fear	82.13	85.86	89.28
All emotions	83.72	80.19	88.64

It can be seen from Table 3 that the recognition accuracy rate of the multi-modality that combines the two information features of voice and video has been improved to a certain extent compared with that of a single-modality. Taking the average recognition accuracy rate for example, the voice, video, and multi-modality are 83.72, 80.19, and 88.64%, respectively. It can be demonstrated that multi-modal fusion can efficiently realize emotion recognition. In addition, for emotions such as disgust and fear, facial expressions in the video can be more reflected than voice. Therefore, the accuracy of emotion recognition of speech features is low, and the accuracy of recognition of disgust is only 58.36%.

### Comparison With Other Methods

**Figure 9:**
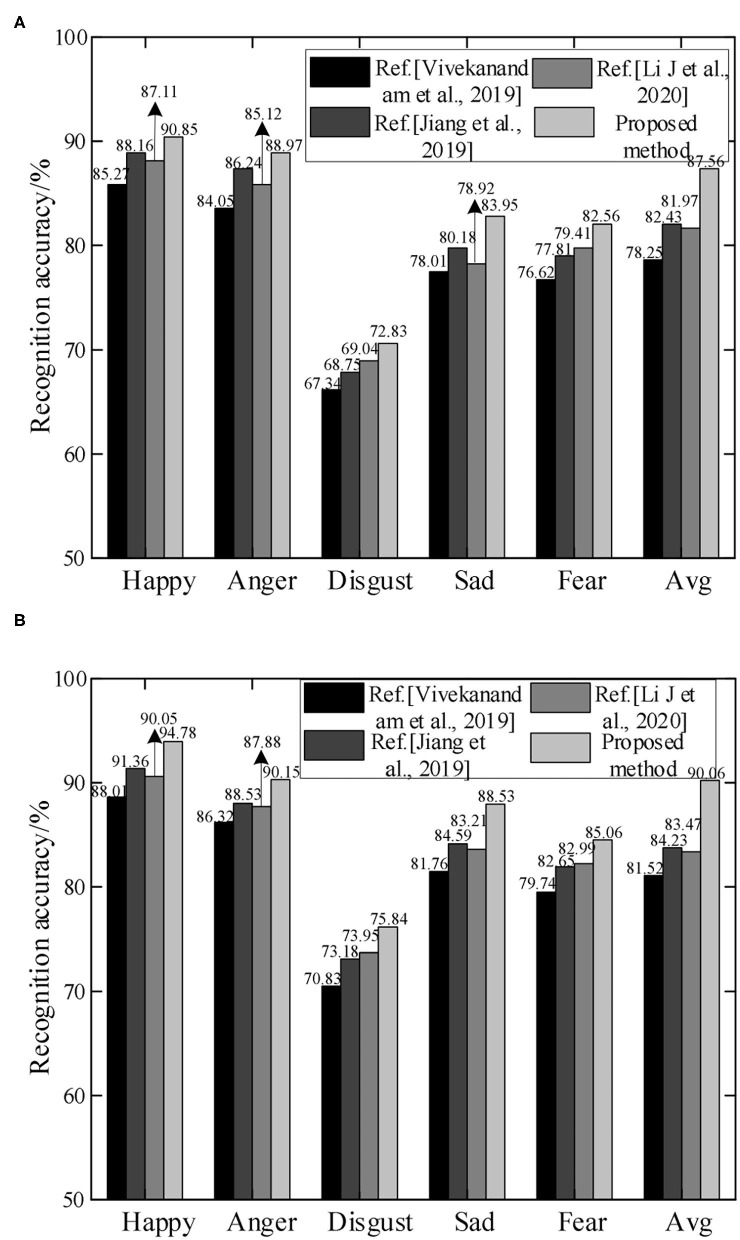
Comparison of recognition accuracy of different methods. **(A)** MOSI dataset and **(B)** MELD dataset.

In order to demonstrate the performance of emotion recognition of the proposed method, it is compared with the methods in Vivekanandam and Babu (2019), Jiang et al. (2019), and (Li et al. (2020) on the MOSI and MELD data sets. The recognition accuracy rates under different emotions are shown in Figure 9.

It can be seen from Figure 9 that compared with other methods, the proposed method has higher recognition accuracy in emotions such as happy, anger, disgust, sad, and fear. Overall, the recognition accuracy rates on the MOSI and MELD datasets are 87.56 and 90.06%, respectively. Since the proposed method adopts two modal features of speech and expression, it can acquire emotional information more fully. At the same time, LIBSVM is used for classification, and the recognition accuracy has been further improved. Vivekanandam and Babu (2019) proposed an HMM model for facial expression classification and recognition in video, and improved random features by Gaussian kernel method. However, the emotion recognition process is mostly based on the ideal environment, and the performance of emotion recognition in complex situations is not good. The average accuracy rate on the MOSI dataset is only 78.25%. Jiang et al. (2019) proposed a parallel convolutional RNN with spectral features for speech emotion recognition, and used the SoftMax classifier for emotion classification. However, single mode still has certain shortcomings in emotion recognition. The recognition accuracy rates in both data sets are lower than 85%. Li et al. (2020) proposed an emotion recognition framework based on RNN with parameter deviation. Emotion recognition is realized by fusing human body joint coordinates, angle and angular velocity to capture expressions. However, the processing power of the learning algorithm needs to be improved. Since the depth of monomodal feature extraction directly affects the accuracy of emotion recognition, the proposed method uses CNN-LSTM network and Inception-ResNet-v2 network to extract the features of facial expressions in voice and video, respectively. After feature selection and fusion, the LIBSVM classifier is input to achieve accurate emotion recognition.

In addition, in the recognition of different emotions, the recognition accuracy on the MELD dataset is slightly higher than that of the MOSI dataset. Because the MELD dataset has richer information, including voice and expression, it is more conducive to model learning. At the same time, due to the similarities among the three emotions of disgust, fear, and sad, the recognition accuracy has been reduced. The proposed method has only 72.83 and 75.84% accuracy in the recognition of disgust emotion on the MOSI and MELD datasets.

## Conclusion

In the context of the era of big data, with the continuous development of artificial intelligence, various modal information has exploded. Aiming at the characteristics of the Internet with multiple modalities, different structures, high dimensions, and large redundant information, a deep learning-based voice expression multi-modal fusion emotion recognition method is proposed. Convolutional neural network-long and short term memory and Inception-Res Net-v2 are used to extract the feature data of facial expressions in voice and video, respectively. At the same time, the acquired modal features are input into the LSTM unit, and the single modalities are spliced to obtain fusion features by the chi-square test feature selection method. And input the output feature data of LSTM into the LIBSVM classifier to realize emotion recognition. Based on the MOSI and MELD datasets, the proposed method is experimentally demonstrated. The results show that when the network weight is about 0.57 and the chi-square test is added, the performance of emotion recognition is the best. And the feature selection ability of the fully connected layer of the Inception-Res Net-v2 network is the best, at 85.75%. Based on the MOSI and MELD data sets, the proposed method is experimentally demonstrated. The results show that the recognition performance is the best when the network weight is about 0.57, and the chi-square test can effectively improve the recognition accuracy. The recognition accuracy of the proposed method on the two data sets are 87.56and 90.06%, respectively, which are superior to other comparison methods and have certain advantages.

The proposed method only considers de-redundancy and noise removal of the internal features of the modal, but in fact, there are some complementary correlations between the information between the modules. In the following research, all single-modal features are mapped to a unified feature space before feature selection. And compare the feature selection within the modal with the feature selection between the modals to get the best multi-modal emotion recognition model structure.

## Data Availability Statement

The original contributions presented in the study are included in the article/supplementary material, further inquiries can be directed to the corresponding author/s.

## Author Contributions

The main idea of this paper is proposed and writing of the article is completed by ZW. The algorithm design and experimental environment construction are completed by LW and LC. The writing guidance, English polish, and funding project are completed by DL. All authors completed experimental verification, contributed to the article, and approved the submitted version.

## Conflict of Interest

The authors declare that the research was conducted in the absence of any commercial or financial relationships that could be construed as a potential conflict of interest.
